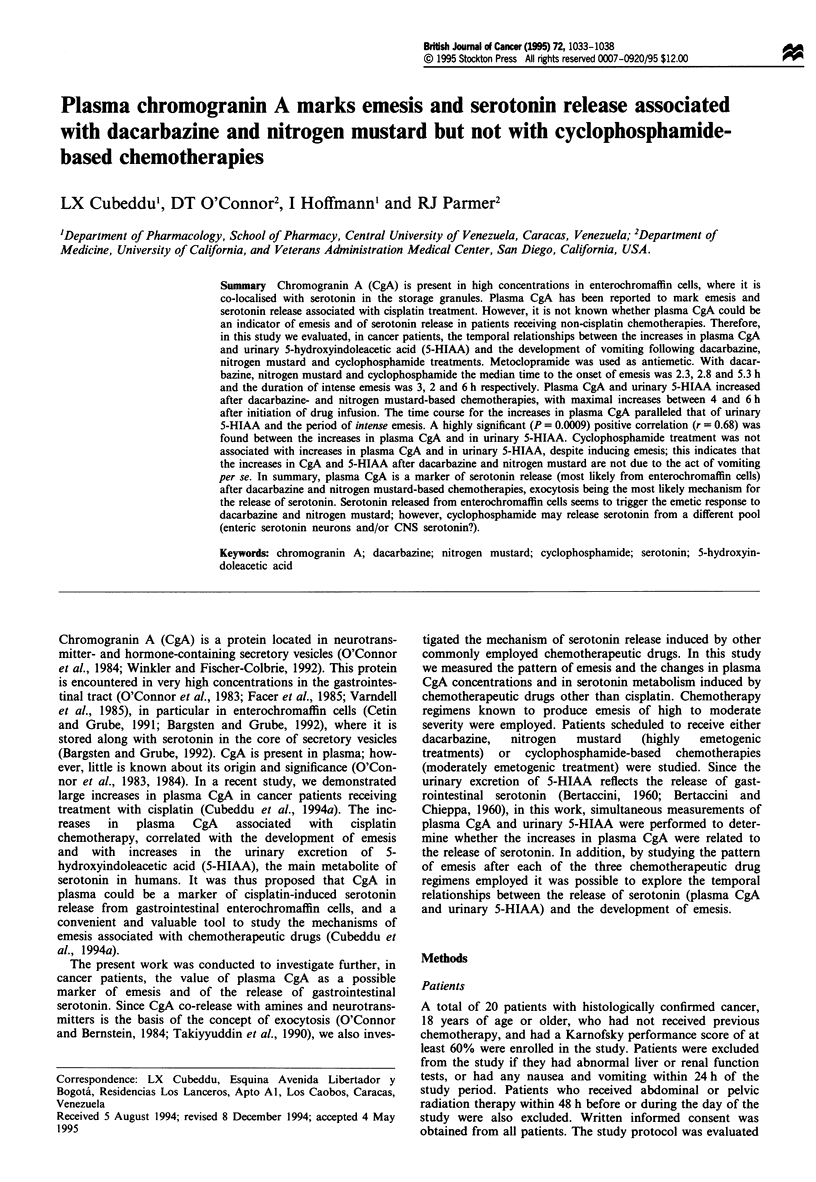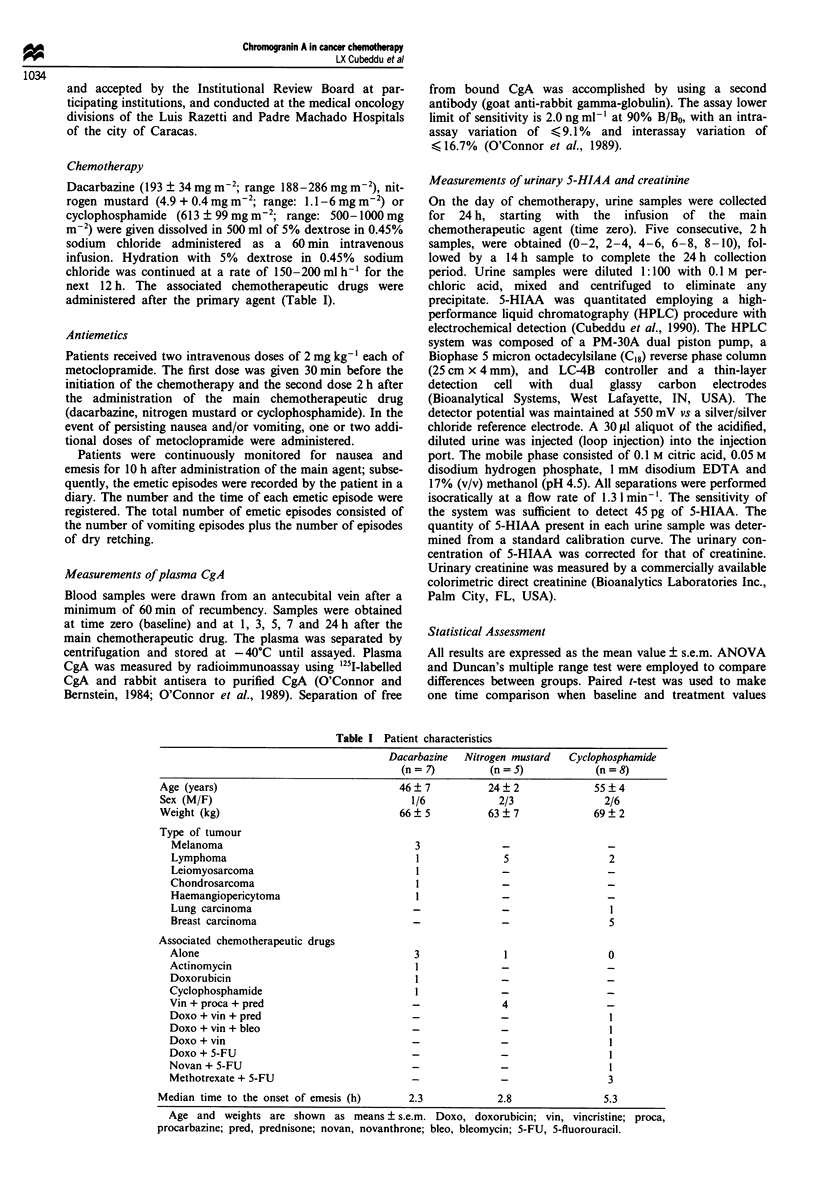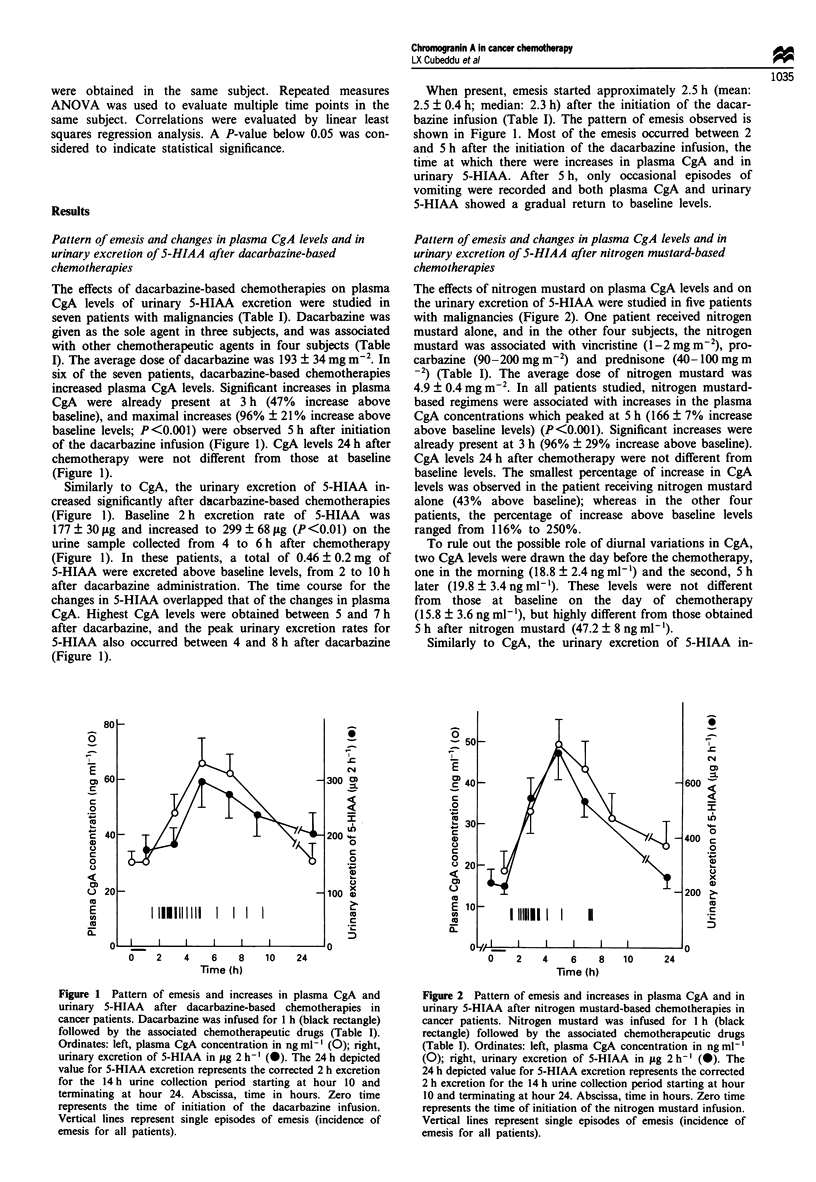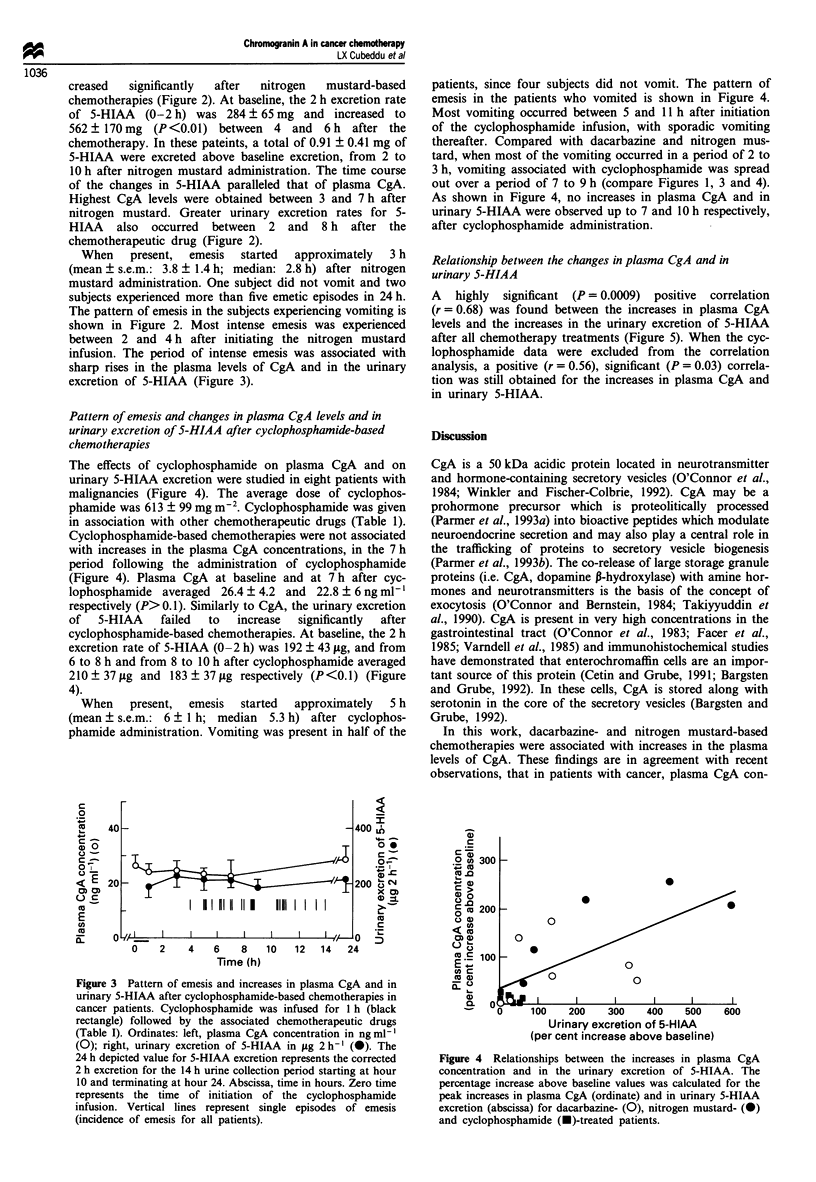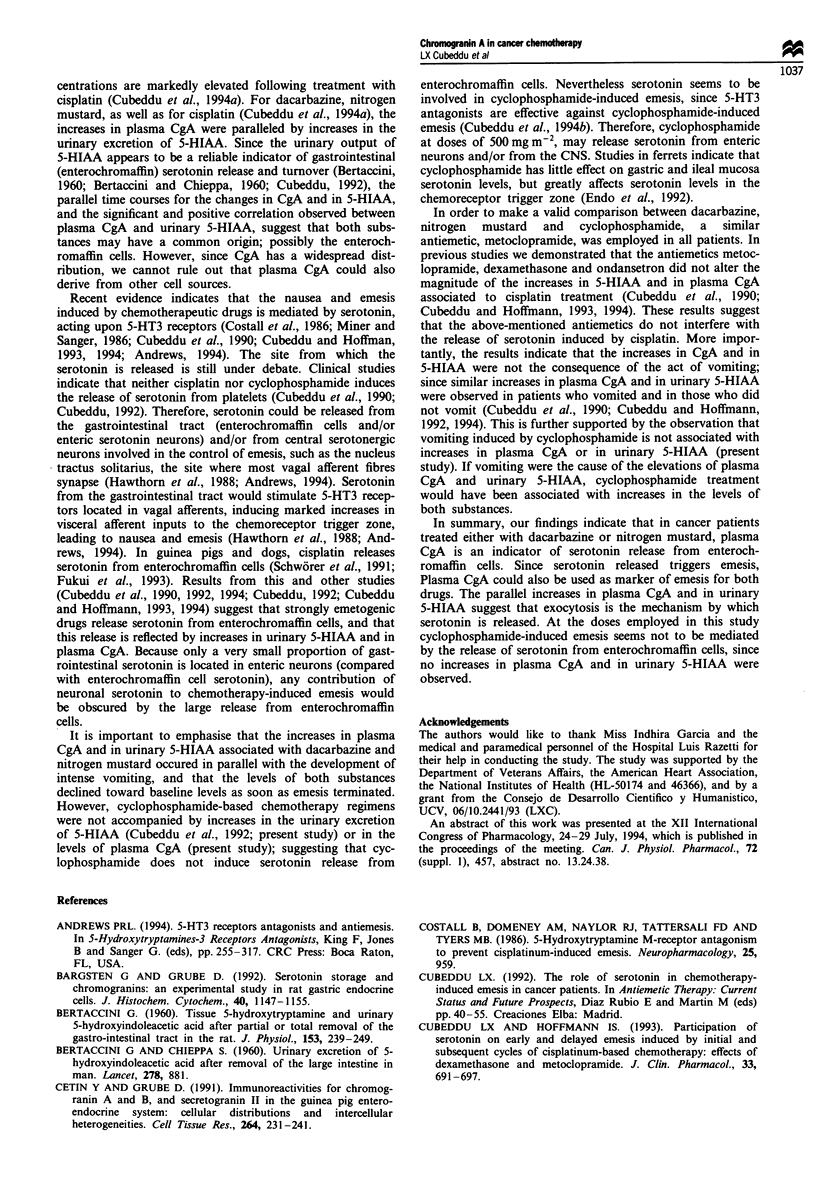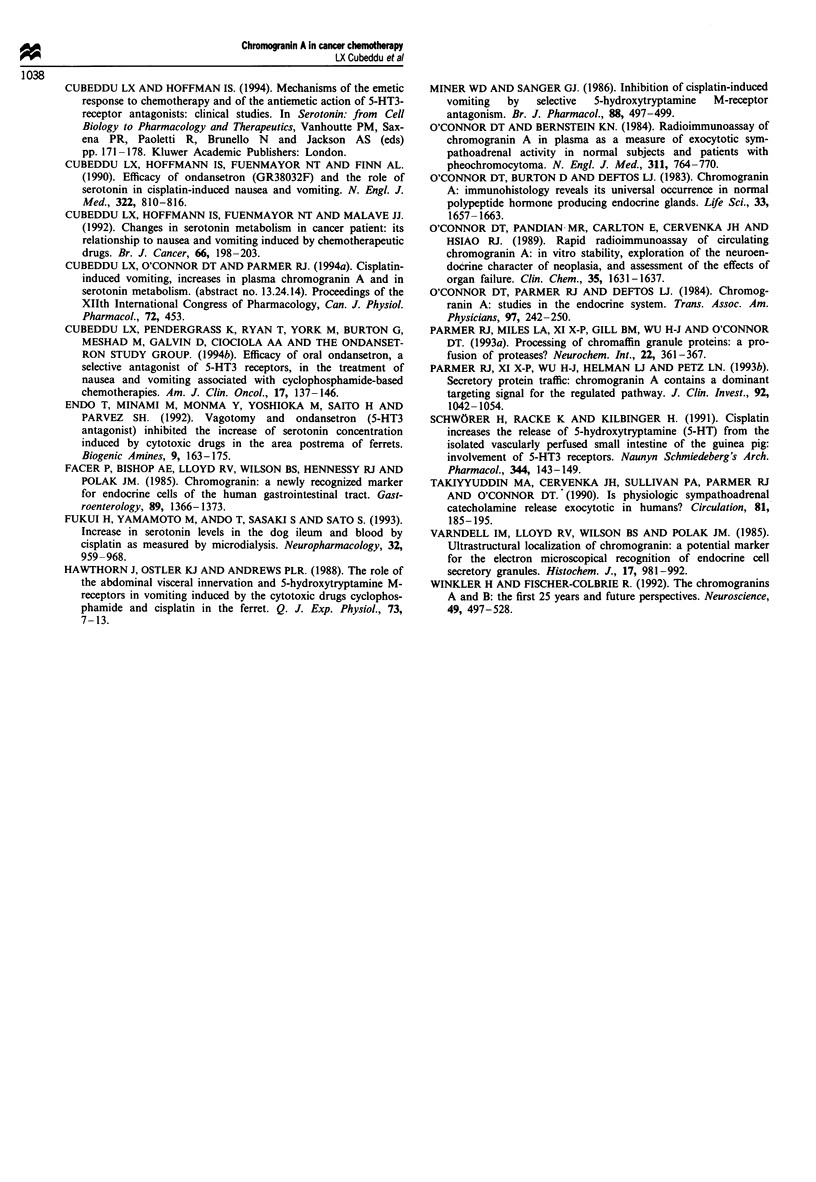# Plasma chromogranin A marks emesis and serotonin release associated with dacarbazine and nitrogen mustard but not with cyclophosphamide-based chemotherapies.

**DOI:** 10.1038/bjc.1995.457

**Published:** 1995-10

**Authors:** L. X. Cubeddu, D. T. O'Connor, I. Hoffmann, R. J. Parmer

**Affiliations:** Department of Pharmacology, School of Pharmacy, Central University of Venezuela, Caracas.

## Abstract

Chromogranin A (CgA) is present in high concentrations in enterochromaffin cells, where it is co-localised with serotonin in the storage granules. Plasma CgA has been reported to mark emesis and serotonin release associated with cisplatin treatment. However, it is not known whether plasma CgA could be an indicator of emesis and of serotonin release in patients receiving non-cisplatin chemotherapies. Therefore, in this study we evaluated, in cancer patients, the temporal relationships between the increases in plasma CgA and urinary 5-hydroxyindoleacetic acid (5-HIAA) and the development of vomiting following dacarbazine, nitrogen mustard and cyclophosphamide treatments. Metoclopramide was used as antiemetic. With dacarbazine, nitrogen mustard and cyclophosphamide the median time to the onset of emesis was 2.3, 2.8 and 5.3 h and the duration of intense emesis was 3, 2 and 6 h respectively. Plasma CgA and urinary 5-HIAA increased after dacarbazine- and nitrogen mustard-based chemotherapies, with maximal increases between 4 and 6 h after initiation of drug infusion. The time course for the increases in plasma CgA paralleled that of urinary 5-HIAA and the period of intense emesis. A highly significant (P = 0.0009) positive correlation (r = 0.68) was found between the increases in plasma CgA and in urinary 5-HIAA. Cyclophosphamide treatment was not associated with increases in plasma CgA and in urinary 5-HIAA, despite inducing emesis; this indicates that the increases in CgA and 5-HIAA after dacarbazine and nitrogen mustard are not due to the act of vomiting per se. In summary, plasma CgA is a marker of serotonin release (most likely from enterochromaffin cells) after dacarbazine and nitrogen mustard-based chemotherapies, exocytosis being the most likely mechanism for the release of serotonin. Serotonin released from enterochromaffin cells seems to trigger the emetic response to dacarbazine and nitrogen mustard; however, cyclophosphamide may release serotonin from a different pool (enteric serotonin neurons and/or CNS serotonin?).


British Journal of Cancer (1995) 72, 1033-1038

© 1995 Stockton Press All rights reserved 0007-0920/95 $12.00

### Plasma chromogranin A marks emesis and serotonin release associated with dacarbazine and nitrogen mustard but not with cyclophosphamide-based chemotherapies

LX Cubeddu[1], DT O'Connor[2], I Hoffmann[1] and RJ Parmer[2]

[1]*Department of Pharmacology, School of Pharmacy, Central University of Venezuela, Caracas, Venezuela;* [2]*Department of Medicine, University of California, and Veterans Administration Medical Center, San Diego, California, USA.*

**Summary** Chromogranin A (CgA) is present in high concentrations in enterochromaffin cells, where it is co-localised with serotonin in the storage granules. Plasma CgA has been reported to mark emesis and serotonin release associated with cisplatin treatment. However, it is not known whether plasma CgA could be an indicator of emesis and of serotonin release in patients receiving non-cisplatin chemotherapies. Therefore, in this study we evaluated, in cancer patients, the temporal relationships between the increases in plasma CgA and urinary 5-hydroxyindoleacetic acid (5-HIAA) and the development of vomiting following dacarbazine, nitrogen mustard and cyclophosphamide treatments. Metoclopramide was used as antiemetic. With dacarbazine, nitrogen mustard and cyclophosphamide the median time to the onset of emesis was 2.3, 2.8 and 5.3 h and the duration of intense emesis was 3, 2 and 6 h respectively. Plasma CgA and urinary 5-HIAA increased after dacarbazine- and nitrogen mustard-based chemotherapies, with maximal increases between 4 and 6 h after initiation of drug infusion. The time course for the increases in plasma CgA paralleled that of urinary 5-HIAA and the period of *intense* emesis. A highly significant ($P = 0.0009$) positive correlation ($r = 0.68$) was found between the increases in plasma CgA and in urinary 5-HIAA. Cyclophosphamide treatment was not associated with increases in plasma CgA and in urinary 5-HIAA, despite inducing emesis; this indicates that the increases in CgA and 5-HIAA after dacarbazine and nitrogen mustard are not due to the act of vomiting *per se*. In summary, plasma CgA is a marker of serotonin release (most likely from enterochromaffin cells) after dacarbazine and nitrogen mustard-based chemotherapies, exocytosis being the most likely mechanism for the release of serotonin. Serotonin released from enterochromaffin cells seems to trigger the emetic response to dacarbazine and nitrogen mustard; however, cyclophosphamide may release serotonin from a different pool (enteric serotonin neurons and/or CNS serotonin?).

**Keywords:** chromogranin A; dacarbazine; nitrogen mustard; cyclophosphamide; serotonin; 5-hydroxyindoleacetic acid

Chromogranin A (CgA) is a protein located in neurotransmitter- and hormone-containing secretory vesicles (O'Connor *et al.*, 1984; Winkler and Fischer-Colbrie, 1992). This protein is encountered in very high concentrations in the gastrointestinal tract (O'Connor *et al.*, 1983; Facer *et al.*, 1985; Varndell *et al.*, 1985), in particular in enterochromaffin cells (Cetin and Grube, 1991; Bargsten and Grube, 1992), where it is stored along with serotonin in the core of secretory vesicles (Bargsten and Grube, 1992). CgA is present in plasma; however, little is known about its origin and significance (O'Connor *et al.*, 1983, 1984). In a recent study, we demonstrated large increases in plasma CgA in cancer patients receiving treatment with cisplatin (Cubeddu *et al.*, 1994*a*). The increases in plasma CgA associated with cisplatin chemotherapy, correlated with the development of emesis and with increases in the urinary excretion of 5-hydroxyindoleacetic acid (5-HIAA), the main metabolite of serotonin in humans. It was thus proposed that CgA in plasma could be a marker of cisplatin-induced serotonin release from gastrointestinal enterochromaffin cells, and a convenient and valuable tool to study the mechanisms of emesis associated with chemotherapeutic drugs (Cubeddu *et al.*, 1994*a*).

The present work was conducted to investigate further, in cancer patients, the value of plasma CgA as a possible marker of emesis and of the release of gastrointestinal serotonin. Since CgA co-release with amines and neurotransmitters is the basis of the concept of exocytosis (O'Connor and Bernstein, 1984; Takiyyuddin *et al.*, 1990), we also investigated the mechanism of serotonin release induced by other commonly employed chemotherapeutic drugs. In this study we measured the pattern of emesis and the changes in plasma CgA concentrations and in serotonin metabolism induced by chemotherapeutic drugs other than cisplatin. Chemotherapy regimens known to produce emesis of high to moderate severity were employed. Patients scheduled to receive either dacarbazine, nitrogen mustard (highly emetogenic treatments) or cyclophosphamide-based chemotherapies (moderately emetogenic treatment) were studied. Since the urinary excretion of 5-HIAA reflects the release of gastrointestinal serotonin (Bertaccini, 1960; Bertaccini and Chieppa, 1960), in this work, simultaneous measurements of plasma CgA and urinary 5-HIAA were performed to determine whether the increases in plasma CgA were related to the release of serotonin. In addition, by studying the pattern of emesis after each of the three chemotherapeutic drug regimens employed it was possible to explore the temporal relationships between the release of serotonin (plasma CgA and urinary 5-HIAA) and the development of emesis.

### Methods

### *Patients*

A total of 20 patients with histologically confirmed cancer, 18 years of age or older, who had not received previous chemotherapy, and had a Karnofsky performance score of at least 60% were enrolled in the study. Patients were excluded from the study if they had abnormal liver or renal function tests, or had any nausea and vomiting within 24 h of the study period. Patients who received abdominal or pelvic radiation therapy within 48 h before or during the day of the study were also excluded. Written informed consent was obtained from all patients. The study protocol was evaluated

Correspondence: LX Cubeddu, Esquina Avenida Libertador y Bogotá, Residencias Los Lanceros, Apto A1, Los Caobos, Caracas, Venezuela
Received 5 August 1994; revised 8 December 1994; accepted 4 May 1995

and accepted by the Institutional Review Board at participating institutions, and conducted at the medical oncology divisions of the Luis Razetti and Padre Machado Hospitals of the city of Caracas.

### *Chemotherapy*

Dacarbazine (193 ± 34 mg $m^{-2}$; range 188–286 mg $m^{-2}$), nitrogen mustard (4.9 + 0.4 mg $m^{-2}$; range: 1.1–6 mg $m^{-2}$) or cyclophosphamide (613 ± 99 mg $m^{-2}$; range: 500–1000 mg $m^{-2}$) were given dissolved in 500 ml of 5% dextrose in 0.45% sodium chloride administered as a 60 min intravenous infusion. Hydration with 5% dextrose in 0.45% sodium chloride was continued at a rate of 150–200 ml $h^{-1}$ for the next 12 h. The associated chemotherapeutic drugs were administered after the primary agent (Table I).

### *Antiemetics*

Patients received two intravenous doses of 2 mg $kg^{-1}$ each of metoclopramide. The first dose was given 30 min before the initiation of the chemotherapy and the second dose 2 h after the administration of the main chemotherapeutic drug (dacarbazine, nitrogen mustard or cyclophosphamide). In the event of persisting nausea and/or vomiting, one or two additional doses of metoclopramide were administered.

Patients were continuously monitored for nausea and emesis for 10 h after administration of the main agent; subsequently, the emetic episodes were recorded by the patient in a diary. The number and the time of each emetic episode were registered. The total number of emetic episodes consisted of the number of vomiting episodes plus the number of episodes of dry retching.

### *Measurements of plasma CgA*

Blood samples were drawn from an antecubital vein after a minimum of 60 min of recumbency. Samples were obtained at time zero (baseline) and at 1, 3, 5, 7 and 24 h after the main chemotherapeutic drug. The plasma was separated by centrifugation and stored at −40°C until assayed. Plasma CgA was measured by radioimmunoassay using $^{125}$I-labelled CgA and rabbit antisera to purified CgA (O'Connor and Bernstein, 1984; O'Connor *et al.*, 1989). Separation of free from bound CgA was accomplished by using a second antibody (goat anti-rabbit gamma-globulin). The assay lower limit of sensitivity is 2.0 ng $ml^{-1}$ at 90% $B/B_0$, with an intra-assay variation of ⩽9.1% and interassay variation of ⩽16.7% (O'Connor *et al.*, 1989).

### *Measurements of urinary 5-HIAA and creatinine*

On the day of chemotherapy, urine samples were collected for 24 h, starting with the infusion of the main chemotherapeutic agent (time zero). Five consecutive, 2 h samples, were obtained (0–2, 2–4, 4–6, 6–8, 8–10), followed by a 14 h sample to complete the 24 h collection period. Urine samples were diluted 1:100 with 0.1 M perchloric acid, mixed and centrifuged to eliminate any precipitate. 5-HIAA was quantitated employing a high-performance liquid chromatography (HPLC) procedure with electrochemical detection (Cubeddu *et al.*, 1990). The HPLC system was composed of a PM-30A dual piston pump, a Biophase 5 micron octadecylsilane ($C_{18}$) reverse phase column (25 cm × 4 mm), and LC-4B controller and a thin-layer detection cell with dual glassy carbon electrodes (Bioanalytical Systems, West Lafayette, IN, USA). The detector potential was maintained at 550 mV *vs* a silver/silver chloride reference electrode. A 30 µl aliquot of the acidified, diluted urine was injected (loop injection) into the injection port. The mobile phase consisted of 0.1 M citric acid, 0.05 M disodium hydrogen phosphate, 1 mM disodium EDTA and 17% (v/v) methanol (pH 4.5). All separations were performed isocratically at a flow rate of 1.3 l $min^{-1}$. The sensitivity of the system was sufficient to detect 45 pg of 5-HIAA. The quantity of 5-HIAA present in each urine sample was determined from a standard calibration curve. The urinary concentration of 5-HIAA was corrected for that of creatinine. Urinary creatinine was measured by a commercially available colorimetric direct creatinine (Bioanalytics Laboratories Inc., Palm City, FL, USA).

### *Statistical Assessment*

All results are expressed as the mean value ± s.e.m. ANOVA and Duncan's multiple range test were employed to compare differences between groups. Paired *t*-test was used to make one time comparison when baseline and treatment values

**Table I** Patient characteristics

| | *Dacarbazine* (n = *7*) | *Nitrogen mustard* (n = *5*) | *Cyclophosphamide* (n = *8*) |
|---|---|---|---|
| Age (years) | 46 ± 7 | 24 ± 2 | 55 ± 4 |
| Sex (M/F) | 1/6 | 2/3 | 2/6 |
| Weight (kg) | 66 ± 5 | 63 ± 7 | 69 ± 2 |
| Type of tumour | | | |
| Melanoma | 3 | – | – |
| Lymphoma | 1 | 5 | 2 |
| Leiomyosarcoma | 1 | – | – |
| Chondrosarcoma | 1 | – | – |
| Haemangiopericytoma | 1 | – | – |
| Lung carcinoma | – | – | 1 |
| Breast carcinoma | – | – | 5 |
| Associated chemotherapeutic drugs | | | |
| Alone | 3 | 1 | 0 |
| Actinomycin | 1 | – | – |
| Doxorubicin | 1 | – | – |
| Cyclophosphamide | 1 | – | – |
| Vin + proca + pred | – | 4 | – |
| Doxo + vin + pred | – | – | 1 |
| Doxo + vin + bleo | – | – | 1 |
| Doxo + vin | – | – | 1 |
| Doxo + 5-FU | – | – | 1 |
| Novan + 5-FU | – | – | 1 |
| Methotrexate + 5-FU | – | – | 3 |
| Median time to the onset of emesis (h) | 2.3 | 2.8 | 5.3 |

Age and weights are shown as means ± s.e.m. Doxo, doxorubicin; vin, vincristine; proca, procarbazine; pred, prednisone; novan, novanthrone; bleo, bleomycin; 5-FU, 5-fluorouracil.

were obtained in the same subject. Repeated measures ANOVA was used to evaluate multiple time points in the same subject. Correlations were evaluated by linear least squares regression analysis. A $P$-value below 0.05 was considered to indicate statistical significance.

### Results

*Pattern of emesis and changes in plasma CgA levels and in urinary excretion of 5-HIAA after dacarbazine-based chemotherapies*

The effects of dacarbazine-based chemotherapies on plasma CgA levels of urinary 5-HIAA excretion were studied in seven patients with malignancies (Table I). Dacarbazine was given as the sole agent in three subjects, and was associated with other chemotherapeutic agents in four subjects (Table I). The average dose of dacarbazine was $193 \pm 34$ mg m$^{-2}$. In six of the seven patients, dacarbazine-based chemotherapies increased plasma CgA levels. Significant increases in plasma CgA were already present at 3 h (47% increase above baseline), and maximal increases (96% $\pm$ 21% increase above baseline levels; $P<0.001$) were observed 5 h after initiation of the dacarbazine infusion (Figure 1). CgA levels 24 h after chemotherapy were not different from those at baseline (Figure 1).

Similarly to CgA, the urinary excretion of 5-HIAA increased significantly after dacarbazine-based chemotherapies (Figure 1). Baseline 2 h excretion rate of 5-HIAA was $177 \pm 30$ μg and increased to $299 \pm 68$ μg ($P<0.01$) on the urine sample collected from 4 to 6 h after chemotherapy (Figure 1). In these patients, a total of $0.46 \pm 0.2$ mg of 5-HIAA were excreted above baseline levels, from 2 to 10 h after dacarbazine administration. The time course for the changes in 5-HIAA overlapped that of the changes in plasma CgA. Highest CgA levels were obtained between 5 and 7 h after dacarbazine, and the peak urinary excretion rates for 5-HIAA also occurred between 4 and 8 h after dacarbazine (Figure 1).

When present, emesis started approximately 2.5 h (mean: $2.5 \pm 0.4$ h; median: 2.3 h) after the initiation of the dacarbazine infusion (Table I). The pattern of emesis observed is shown in Figure 1. Most of the emesis occurred between 2 and 5 h after the initiation of the dacarbazine infusion, the time at which there were increases in plasma CgA and in urinary 5-HIAA. After 5 h, only occasional episodes of vomiting were recorded and both plasma CgA and urinary 5-HIAA showed a gradual return to baseline levels.

*Pattern of emesis and changes in plasma CgA levels and in urinary excretion of 5-HIAA after nitrogen mustard-based chemotherapies*

The effects of nitrogen mustard on plasma CgA levels and on the urinary excretion of 5-HIAA were studied in five patients with malignancies (Figure 2). One patient received nitrogen mustard alone, and in the other four subjects, the nitrogen mustard was associated with vincristine (1–2 mg m$^{-2}$), procarbazine (90–200 mg m$^{-2}$) and prednisone (40–100 mg m $^{-2}$) (Table I). The average dose of nitrogen mustard was $4.9 \pm 0.4$ mg m$^{-2}$. In all patients studied, nitrogen mustard-based regimens were associated with increases in the plasma CgA concentrations which peaked at 5 h ($166 \pm 7$% increase above baseline levels) ($P<0.001$). Significant increases were already present at 3 h (96% $\pm$ 29% increase above baseline). CgA levels 24 h after chemotherapy were not different from baseline levels. The smallest percentage of increase in CgA levels was observed in the patient receiving nitrogen mustard alone (43% above baseline); whereas in the other four patients, the percentage of increase above baseline levels ranged from 116% to 250%.

To rule out the possible role of diurnal variations in CgA, two CgA levels were drawn the day before the chemotherapy, one in the morning ($18.8 \pm 2.4$ ng ml$^{-1}$) and the second, 5 h later ($19.8 \pm 3.4$ ng ml$^{-1}$). These levels were not different from those at baseline on the day of chemotherapy ($15.8 \pm 3.6$ ng ml$^{-1}$), but highly different from those obtained 5 h after nitrogen mustard ($47.2 \pm 8$ ng ml$^{-1}$).

Similarly to CgA, the urinary excretion of 5-HIAA in-

**Figure 1** Pattern of emesis and increases in plasma CgA and urinary 5-HIAA after dacarbazine-based chemotherapies in cancer patients. Dacarbazine was infused for 1 h (black rectangle) followed by the associated chemotherapeutic drugs (Table I). Ordinates: left, plasma CgA concentration in ng ml$^{-1}$ (○); right, urinary excretion of 5-HIAA in μg 2 h$^{-1}$ (●). The 24 h depicted value for 5-HIAA excretion represents the corrected 2 h excretion for the 14 h urine collection period starting at hour 10 and terminating at hour 24. Abscissa, time in hours. Zero time represents the time of initiation of the dacarbazine infusion. Vertical lines represent single episodes of emesis (incidence of emesis for all patients).

**Figure 2** Pattern of emesis and increases in plasma CgA and in urinary 5-HIAA after nitrogen mustard-based chemotherapies in cancer patients. Nitrogen mustard was infused for 1 h (black rectangle) followed by the associated chemotherapeutic drugs (Table I). Ordinates: left, plasma CgA concentration in ng ml$^{-1}$ (○); right, urinary excretion of 5-HIAA in μg 2 h$^{-1}$ (●). The 24 h depicted value for 5-HIAA excretion represents the corrected 2 h excretion for the 14 h urine collection period starting at hour 10 and terminating at hour 24. Abscissa, time in hours. Zero time represents the time of initiation of the nitrogen mustard infusion. Vertical lines represent single episodes of emesis (incidence of emesis for all patients).

creased significantly after nitrogen mustard-based chemotherapies (Figure 2). At baseline, the 2 h excretion rate of 5-HIAA (0–2 h) was 284 ± 65 mg and increased to 562 ± 170 mg ($P$<0.01) between 4 and 6 h after the chemotherapy. In these pateints, a total of 0.91 ± 0.41 mg of 5-HIAA were excreted above baseline excretion, from 2 to 10 h after nitrogen mustard administration. The time course of the changes in 5-HIAA paralleled that of plasma CgA. Highest CgA levels were obtained between 3 and 7 h after nitrogen mustard. Greater urinary excretion rates for 5-HIAA also occurred between 2 and 8 h after the chemotherapeutic drug (Figure 2).

When present, emesis started approximately 3 h (mean ± s.e.m.: 3.8 ± 1.4 h; median: 2.8 h) after nitrogen mustard administration. One subject did not vomit and two subjects experienced more than five emetic episodes in 24 h. The pattern of emesis in the subjects experiencing vomiting is shown in Figure 2. Most intense emesis was experienced between 2 and 4 h after initiating the nitrogen mustard infusion. The period of intense emesis was associated with sharp rises in the plasma levels of CgA and in the urinary excretion of 5-HIAA (Figure 3).

*Pattern of emesis and changes in plasma CgA levels and in urinary excretion of 5-HIAA after cyclophosphamide-based chemotherapies*

The effects of cyclophosphamide on plasma CgA and on urinary 5-HIAA excretion were studied in eight patients with malignancies (Figure 4). The average dose of cyclophosphamide was 613 ± 99 mg m$^{-2}$. Cyclophosphamide was given in association with other chemotherapeutic drugs (Table 1). Cyclophosphamide-based chemotherapies were not associated with increases in the plasma CgA concentrations, in the 7 h period following the administration of cyclophosphamide (Figure 4). Plasma CgA at baseline and at 7 h after cyclophosphamide averaged 26.4 ± 4.2 and 22.8 ± 6 ng ml$^{-1}$ respectively ($P$> 0.1). Similarly to CgA, the urinary excretion of 5-HIAA failed to increase significantly after cyclophosphamide-based chemotherapies. At baseline, the 2 h excretion rate of 5-HIAA (0–2 h) was 192 ± 43 μg, and from 6 to 8 h and from 8 to 10 h after cyclophosphamide averaged 210 ± 37 μg and 183 ± 37 μg respectively ($P$<0.1) (Figure 4).

When present, emesis started approximately 5 h (mean ± s.e.m.: 6 ± 1 h; median 5.3 h) after cyclophosphamide administration. Vomiting was present in half of the patients, since four subjects did not vomit. The pattern of emesis in the patients who vomited is shown in Figure 4. Most vomiting occurred between 5 and 11 h after initiation of the cyclophosphamide infusion, with sporadic vomiting thereafter. Compared with dacarbazine and nitrogen mustard, when most of the vomiting occurred in a period of 2 to 3 h, vomiting associated with cyclophosphamide was spread out over a period of 7 to 9 h (compare Figures 1, 3 and 4). As shown in Figure 4, no increases in plasma CgA and in urinary 5-HIAA were observed up to 7 and 10 h respectively, after cyclophosphamide administration.

*Relationship between the changes in plasma CgA and in urinary 5-HIAA*

A highly significant ($P$ = 0.0009) positive correlation ($r$ = 0.68) was found between the increases in plasma CgA levels and the increases in the urinary excretion of 5-HIAA after all chemotherapy treatments (Figure 5). When the cyclophosphamide data were excluded from the correlation analysis, a positive ($r$ = 0.56), significant ($P$ = 0.03) correlation was still obtained for the increases in plasma CgA and in urinary 5-HIAA.

### Discussion

CgA is a 50 kDa acidic protein located in neurotransmitter and hormone-containing secretory vesicles (O'Connor *et al.*, 1984; Winkler and Fischer-Colbrie, 1992). CgA may be a prohormone precursor which is proteolitically processed (Parmer *et al.*, 1993*a*) into bioactive peptides which modulate neuroendocrine secretion and may also play a central role in the trafficking of proteins to secretory vesicle biogenesis (Parmer *et al.*, 1993*b*). The co-release of large storage granule proteins (i.e. CgA, dopamine β-hydroxylase) with amine hormones and neurotransmitters is the basis of the concept of exocytosis (O'Connor and Bernstein, 1984; Takiyyuddin *et al.*, 1990). CgA is present in very high concentrations in the gastrointestinal tract (O'Connor *et al.*, 1983; Facer *et al.*, 1985; Varndell *et al.*, 1985) and immunohistochemical studies have demonstrated that enterochromaffin cells are an important source of this protein (Cetin and Grube, 1991; Bargsten and Grube, 1992). In these cells, CgA is stored along with serotonin in the core of the secretory vesicles (Bargsten and Grube, 1992).

In this work, dacarbazine- and nitrogen mustard-based chemotherapies were associated with increases in the plasma levels of CgA. These findings are in agreement with recent observations, that in patients with cancer, plasma CgA con-

**Figure 3** Pattern of emesis and increases in plasma CgA and in urinary 5-HIAA after cyclophosphamide-based chemotherapies in cancer patients. Cyclophosphamide was infused for 1 h (black rectangle) followed by the associated chemotherapeutic drugs (Table I). Ordinates: left, plasma CgA concentration in ng ml$^{-1}$ (○); right, urinary excretion of 5-HIAA in μg 2 h$^{-1}$ (●). The 24 h depicted value for 5-HIAA excretion represents the corrected 2 h excretion for the 14 h urine collection period starting at hour 10 and terminating at hour 24. Abscissa, time in hours. Zero time represents the time of initiation of the cyclophosphamide infusion. Vertical lines represent single episodes of emesis (incidence of emesis for all patients).

**Figure 4** Relationships between the increases in plasma CgA concentration and in the urinary excretion of 5-HIAA. The percentage increase above baseline values was calculated for the peak increases in plasma CgA (ordinate) and in urinary 5-HIAA excretion (abscissa) for dacarbazine- (○), nitrogen mustard- (●) and cyclophosphamide (■)-treated patients.

centrations are markedly elevated following treatment with cisplatin (Cubeddu *et al.*, 1994*a*). For dacarbazine, nitrogen mustard, as well as for cisplatin (Cubeddu *et al.*, 1994*a*), the increases in plasma CgA were paralleled by increases in the urinary excretion of 5-HIAA. Since the urinary output of 5-HIAA appears to be a reliable indicator of gastrointestinal (enterochromaffin) serotonin release and turnover (Bertaccini, 1960; Bertaccini and Chieppa, 1960; Cubeddu, 1992), the parallel time courses for the changes in CgA and in 5-HIAA, and the significant and positive correlation observed between plasma CgA and urinary 5-HIAA, suggest that both substances may have a common origin; possibly the enterochromaffin cells. However, since CgA has a widespread distribution, we cannot rule out that plasma CgA could also derive from other cell sources.

Recent evidence indicates that the nausea and emesis induced by chemotherapeutic drugs is mediated by serotonin, acting upon 5-HT3 receptors (Costall *et al.*, 1986; Miner and Sanger, 1986; Cubeddu *et al.*, 1990; Cubeddu and Hoffman, 1993, 1994; Andrews, 1994). The site from which the serotonin is released is still under debate. Clinical studies indicate that neither cisplatin nor cyclophosphamide induces the release of serotonin from platelets (Cubeddu *et al.*, 1990; Cubeddu, 1992). Therefore, serotonin could be released from the gastrointestinal tract (enterochromaffin cells and/or enteric serotonin neurons) and/or from central serotonergic neurons involved in the control of emesis, such as the nucleus tractus solitarius, the site where most vagal afferent fibres synapse (Hawthorn *et al.*, 1988; Andrews, 1994). Serotonin from the gastrointestinal tract would stimulate 5-HT3 receptors located in vagal afferents, inducing marked increases in visceral afferent inputs to the chemoreceptor trigger zone, leading to nausea and emesis (Hawthorn *et al.*, 1988; Andrews, 1994). In guinea pigs and dogs, cisplatin releases serotonin from enterochromaffin cells (Schwörer *et al.*, 1991; Fukui *et al.*, 1993). Results from this and other studies (Cubeddu *et al.*, 1990, 1992, 1994; Cubeddu, 1992; Cubeddu and Hoffmann, 1993, 1994) suggest that strongly emetogenic drugs release serotonin from enterochromaffin cells, and that this release is reflected by increases in urinary 5-HIAA and in plasma CgA. Because only a very small proportion of gastrointestinal serotonin is located in enteric neurons (compared with enterochromaffin cell serotonin), any contribution of neuronal serotonin to chemotherapy-induced emesis would be obscured by the large release from enterochromaffin cells.

It is important to emphasise that the increases in plasma CgA and in urinary 5-HIAA associated with dacarbazine and nitrogen mustard occured in parallel with the development of intense vomiting, and that the levels of both substances declined toward baseline levels as soon as emesis terminated. However, cyclophosphamide-based chemotherapy regimens were not accompanied by increases in the urinary excretion of 5-HIAA (Cubeddu *et al.*, 1992; present study) or in the levels of plasma CgA (present study); suggesting that cyclophosphamide does not induce serotonin release from enterochromaffin cells. Nevertheless serotonin seems to be involved in cyclophosphamide-induced emesis, since 5-HT3 antagonists are effective against cyclophosphamide-induced emesis (Cubeddu *et al.*, 1994*b*). Therefore, cyclophosphamide at doses of 500 mg $m^{-2}$, may release serotonin from enteric neurons and/or from the CNS. Studies in ferrets indicate that cyclophosphamide has little effect on gastric and ileal mucosa serotonin levels, but greatly affects serotonin levels in the chemoreceptor trigger zone (Endo *et al.*, 1992).

In order to make a valid comparison between dacarbazine, nitrogen mustard and cyclophosphamide, a similar antiemetic, metoclopramide, was employed in all patients. In previous studies we demonstrated that the antiemetics metoclopramide, dexamethasone and ondansetron did not alter the magnitude of the increases in 5-HIAA and in plasma CgA associated to cisplatin treatment (Cubeddu *et al.*, 1990; Cubeddu and Hoffmann, 1993, 1994). These results suggest that the above-mentioned antiemetics do not interfere with the release of serotonin induced by cisplatin. More importantly, the results indicate that the increases in CgA and in 5-HIAA were not the consequence of the act of vomiting; since similar increases in plasma CgA and in urinary 5-HIAA were observed in patients who vomited and in those who did not vomit (Cubeddu *et al.*, 1990; Cubeddu and Hoffmann, 1992, 1994). This is further supported by the observation that vomiting induced by cyclophosphamide is not associated with increases in plasma CgA or in urinary 5-HIAA (present study). If vomiting were the cause of the elevations of plasma CgA and urinary 5-HIAA, cyclophosphamide treatment would have been associated with increases in the levels of both substances.

In summary, our findings indicate that in cancer patients treated either with dacarbazine or nitrogen mustard, plasma CgA is an indicator of serotonin release from enterochromaffin cells. Since serotonin released triggers emesis, Plasma CgA could also be used as marker of emesis for both drugs. The parallel increases in plasma CgA and in urinary 5-HIAA suggest that exocytosis is the mechanism by which serotonin is released. At the doses employed in this study cyclophosphamide-induced emesis seems not to be mediated by the release of serotonin from enterochromaffin cells, since no increases in plasma CgA and in urinary 5-HIAA were observed.

**Acknowledgements**
The authors would like to thank Miss Indhira Garcia and the medical and paramedical personnel of the Hospital Luis Razetti for their help in conducting the study. The study was supported by the Department of Veterans Affairs, the American Heart Association, the National Institutes of Health (HL-50174 and 46366), and by a grant from the Consejo de Desarrollo Cientifico y Humanistico, UCV, 06/10.2441/93 (LXC).

An abstract of this work was presented at the XII International Congress of Pharmacology, 24–29 July, 1994, which is published in the proceedings of the meeting. *Can. J. Physiol. Pharmacol.*, **72** (suppl. 1), 457, abstract no. 13.24.38.

### References

ANDREWS PRL. (1994). 5-HT3 receptors antagonists and antiemesis. In *5-Hydroxytryptamines-3 Receptors Antagonists*, King F, Jones B and Sanger G. (eds), pp. 255–317. CRC Press: Boca Raton, FL, USA.

BARGSTEN G AND GRUBE D. (1992). Serotonin storage and chromogranins: an experimental study in rat gastric endocrine cells. *J. Histochem. Cytochem.*, **40**, 1147–1155.

BERTACCINI G. (1960). Tissue 5-hydroxytryptamine and urinary 5-hydroxyindoleacetic acid after partial or total removal of the gastro-intestinal tract in the rat. *J. Physiol.*, **153**, 239–249.

BERTACCINI G AND CHIEPPA S. (1960). Urinary excretion of 5-hydroxyindoleacetic acid after removal of the large intestine in man. *Lancet*, **278**, 881.

CETIN Y AND GRUBE D. (1991). Immunoreactivities for chromogranin A and B, and secretogranin II in the guinea pig entero-endocrine system: cellular distributions and intercellular heterogeneities. *Cell Tissue Res.*, **264**, 231–241.

COSTALL B, DOMENEY AM, NAYLOR RJ, TATTERSALI FD AND TYERS MB. (1986). 5-Hydroxytryptamine M-receptor antagonism to prevent cisplatinum-induced emesis. *Neuropharmacology*, **25**, 959.

CUBEDDU LX. (1992). The role of serotonin in chemotherapy-induced emesis in cancer patients. In *Antiemetic Therapy: Current Status and Future Prospects*, Diaz Rubio E and Martin M (eds) pp. 40–55. Creaciones Elba: Madrid.

CUBEDDU LX AND HOFFMANN IS. (1993). Participation of serotonin on early and delayed emesis induced by initial and subsequent cycles of cisplatinum-based chemotherapy: effects of dexamethasone and metoclopramide. *J. Clin. Pharmacol.*, **33**, 691–697.

CUBEDDU LX AND HOFFMAN IS. (1994). Mechanisms of the emetic response to chemotherapy and of the antiemetic action of 5-HT3-receptor antagonists: clinical studies. In *Serotonin: from Cell Biology to Pharmacology and Therapeutics*, Vanhoutte PM, Saxena PR, Paoletti R, Brunello N and Jackson AS (eds) pp. 171–178. Kluwer Academic Publishers: London.

CUBEDDU LX, HOFFMANN IS, FUENMAYOR NT AND FINN AL. (1990). Efficacy of ondansetron (GR38032F) and the role of serotonin in cisplatin-induced nausea and vomiting. *N. Engl. J. Med.*, **322,** 810–816.

CUBEDDU LX, HOFFMANN IS, FUENMAYOR NT AND MALAVE JJ. (1992). Changes in serotonin metabolism in cancer patient: its relationship to nausea and vomiting induced by chemotherapeutic drugs. *Br. J. Cancer*, **66,** 198–203.

CUBEDDU LX, O'CONNOR DT AND PARMER RJ. (1994*a*). Cisplatin-induced vomiting, increases in plasma chromogranin A and in serotonin metabolism. (abstract no. 13.24.14). Proceedings of the XIIth International Congress of Pharmacology, *Can. J. Physiol. Pharmacol.*, **72,** 453.

CUBEDDU LX, PENDERGRASS K, RYAN T, YORK M, BURTON G, MESHAD M, GALVIN D, CIOCIOLA AA AND THE ONDANSETRON STUDY GROUP. (1994*b*). Efficacy of oral ondansetron, a selective antagonist of 5-HT3 receptors, in the treatment of nausea and vomiting associated with cyclophosphamide-based chemotherapies. *Am. J. Clin. Oncol.*, **17,** 137–146.

ENDO T, MINAMI M, MONMA Y, YOSHIOKA M, SAITO H AND PARVEZ SH. (1992). Vagotomy and ondansetron (5-HT3 antagonist) inhibited the increase of serotonin concentration induced by cytotoxic drugs in the area postrema of ferrets. *Biogenic Amines*, **9,** 163–175.

FACER P, BISHOP AE, LLOYD RV, WILSON BS, HENNESSY RJ AND POLAK JM. (1985). Chromogranin: a newly recognized marker for endocrine cells of the human gastrointestinal tract. *Gastroenterology*, **89,** 1366–1373.

FUKUI H, YAMAMOTO M, ANDO T, SASAKI S AND SATO S. (1993). Increase in serotonin levels in the dog ileum and blood by cisplatin as measured by microdialysis. *Neuropharmacology*, **32,** 959–968.

HAWTHORN J, OSTLER KJ AND ANDREWS PLR. (1988). The role of the abdominal visceral innervation and 5-hydroxytryptamine M-receptors in vomiting induced by the cytotoxic drugs cyclophosphamide and cisplatin in the ferret. *Q. J. Exp. Physiol.*, **73,** 7–13.

MINER WD AND SANGER GJ. (1986). Inhibition of cisplatin-induced vomiting by selective 5-hydroxytryptamine M-receptor antagonism. *Br. J. Pharmacol.*, **88,** 497–499.

O'CONNOR DT AND BERNSTEIN KN. (1984). Radioimmunoassay of chromogranin A in plasma as a measure of exocytotic sympathoadrenal activity in normal subjects and patients with pheochromocytoma. *N. Engl. J. Med.*, **311,** 764–770.

O'CONNOR DT, BURTON D AND DEFTOS LJ. (1983). Chromogranin A: immunohistology reveals its universal occurrence in normal polypeptide hormone producing endocrine glands. *Life Sci.*, **33,** 1657–1663.

O'CONNOR DT, PANDIAN MR, CARLTON E, CERVENKA JH AND HSIAO RJ. (1989). Rapid radioimmunoassay of circulating chromogranin A: in vitro stability, exploration of the neuroendocrine character of neoplasia, and assessment of the effects of organ failure. *Clin. Chem.*, **35,** 1631–1637.

O'CONNOR DT, PARMER RJ AND DEFTOS LJ. (1984). Chromogranin A: studies in the endocrine system. *Trans. Assoc. Am. Physicians*, **97,** 242–250.

PARMER RJ, MILES LA, XI X-P, GILL BM, WU H-J AND O'CONNOR DT. (1993*a*). Processing of chromaffin granule proteins: a profusion of proteases? *Neurochem. Int.*, **22,** 361–367.

PARMER RJ, XI X-P, WU H-J, HELMAN LJ AND PETZ LN. (1993*b*). Secretory protein traffic: chromogranin A contains a dominant targeting signal for the regulated pathway. *J. Clin. Invest.*, **92,** 1042–1054.

SCHWÖRER H, RACKE K AND KILBINGER H. (1991). Cisplatin increases the release of 5-hydroxytryptamine (5-HT) from the isolated vascularly perfused small intestine of the guinea pig: involvement of 5-HT3 receptors. *Naunyn Schmiedeberg's Arch. Pharmacol.*, **344,** 143–149.

TAKIYYUDDIN MA, CERVENKA JH, SULLIVAN PA, PARMER RJ AND O'CONNOR DT. (1990). Is physiologic sympathoadrenal catecholamine release exocytotic in humans? *Circulation*, **81,** 185–195.

VARNDELL IM, LLOYD RV, WILSON BS AND POLAK JM. (1985). Ultrastructural localization of chromogranin: a potential marker for the electron microscopical recognition of endocrine cell secretory granules. *Histochem. J.*, **17,** 981–992.

WINKLER H AND FISCHER-COLBRIE R. (1992). The chromogranins A and B: the first 25 years and future perspectives. *Neuroscience*, **49,** 497–528.